# The effects of Energised Greens™ upon blood acid-base balance during resting conditions

**DOI:** 10.1186/1550-2783-8-14

**Published:** 2011-10-12

**Authors:** Mark Turner, Richard Page, Nigel Mitchell, Jason Siegler

**Affiliations:** 1 Department of Sport, Health and Exercise Science, University of Hull, Hull, UK; 2 Head of Nutrition, British Cycling, Manchester, UK; 3 School of Biomedical and Health Sciences, University of Western Sydney, Penrith, Australia

## Abstract

**Background:**

The consumption of fresh fruit & vegetable in concentrate form (FVC) have recently become an alternative approach to combating excessive renal acid loads often associated with *Western Diets*. Additionally, these FVC's have been purported to induce metabolic alkalosis, which perhaps may enhance the blood buffering capacity of an individual. Therefore, the aim of this preliminary study was to profile the acid-base response after ingestion of an acute dose of fruit and vegetable extract (Energised Greens™ (EG), Nottingham, UK) and compare it to a standard, low dose (0.1 g·kg^-1^) of sodium bicarbonate (NaHCO_3_).

**Findings:**

As part of a randomized, cross over design participants consumed 750 mL of water with either 9 g of EG (manufacturer recommendations), 0.1 g·kg^-1 ^of NaHCO_3 _or a placebo (plain flour) in opaque encapsulated pills following an overnight fast. Capillary samples were obtained and analyzed every 15 min for a period of 120 min following ingestion. Significant interactions (p < 0.01), main effects for condition (p < 0.001) and time (p < 0.001) were evident for all acid-base variables (pH, HCO_3^-^_, BE). Interactions indicated significant elevation in blood alkalosis for only the NaHCO_3 _condition when compared to both placebo and EG from 15 to 120 minutes.

**Conclusions:**

Despite previous findings of elevated blood pH following acute mineral supplementation, manufacturer recommended doses of EG do not induce any significant changes in acid-base regulation in resting males.

## Background

The practice of manipulating acid-base balance for purposes of improving performance has been on going for nearly a century [1]. However, enhancing blood buffering capacity generally requires high acute loads of alkaline substances (e.g. sodium bicarbonate (NaHCO_3_), sodium citrate (C_6_H_5_Na_3_O_7_) or sodium lactate (C_3_H_5_NaO_3_)) that generally place a great deal of stress on the gastrointestinal (GI) system [2]. The prospective negative implications of such a response often push athletes away from using these supplements. The potential for manipulating acid-base balance acutely using alternative strategies, such as through the high alkali-forming nature of certain food extracts (fruit and vegetables) in replace of such buffers is warranted, particularly if the claims of improving alkalinity are indeed true [3]. Traditionally, fruit and vegetable extracts have been used to provide the body with additional (or supplemental) vitamins and minerals to combat excessive renal acid loads often associated with *Western Diets*. By alkalizing the internal milieu, proponents have claimed this approach improves gastric motility, digestion and vitamin and mineral absorption when compared to the acidic western diet [3-5]. With specific reference to inducing metabolic alkalosis, these extracts generally contain high levels of ions recognized for their alkalinizing properties (e.g. citrate which is ultimately metabolized to bicarbonate) [5]. However, the extent to which acute or chronic consumption of these extracts influences blood alkalinity, and ultimately whether or not the relative shift towards metabolic alkalosis substantially alters blood buffering capacity, has not been investigated.

Although the acute effects of fruit and vegetable extracts upon blood buffering capacity have not been researched per se, recently König et al. has investigated the effect of acute multi-mineral supplementation upon both blood and urine pH [3]. These authors indicated a pronounced increase in blood pH three to four hours after supplementation. Other research has documented similar increases in urinary pH following three weeks of prolonged phytonutrient supplementation [6]. Collectively, these investigations illustrate the need for further comparison between alternative (e.g. fruit & vegetable extracts) and traditional (e.g. sodium bicarbonate) strategies used to induce metabolic alkalosis and enhance buffering capacity in order to provide insight into the potential efficacy for using this supplement in a sporting context. Therefore, the aim of this preliminary study was to profile the acid-base response after ingestion of a manufacturer recommended, acute dose of fruit and vegetable extract and compare that to a low, standard dose (0.1 g·kg^-1^BW) of sodium bicarbonate. The fruit and vegetable extract selected for the current study (Energised Greens™) was based upon two factors; 1) the intent of selecting a commercially available product for the purpose of improving the ecological validity of the study and 2) the composition of the extract as indicated by the manufacturer (Table 1) was advertised as an alkali http://www.ayurveda4life.co.uk.

**Table 1:** Energised Greens™ composition provided on manufacturer's label

Ingredients	Per Dose (9 g)
13:1 extract organic whole leaf 13:1 barley grass	4220 mg

Fruits and Greens (*concentrate 100:1 extract from 27 different fruit and vegetables*)	1150 mg

Chlorella (*containing 30 mg·g Chlorophyll*)	300 mg

Spirulina	700 mg

Enzyme Complex (*Fermented Rice*)	40 mg

Fibre Complex (*apple fibre, apple pectin, microironized wheat germ, wheat bran and acacia fibre*)	2000 mg

Lactospore culture (*probiotic stomach acid resistant culture*)	90 mg/1.49 billion

Policosanol Complex	40 mg

Acerola extract (*with 50% vitamin C*)	150 mg

Green tea extract (*40% catechins*)	70 mg

Natural fruit-based aromas	240 mg

## Methods

Eight apparently healthy, recreationally trained males (Age: 23 ± 2 yr; Height: 180.1 ± 6.2 cm; Weight: 76.9 ± 7.2 kg) volunteered to participate in the study. All participants refrained from supplementation of all kinds (i.e., vitamins, ergogenic aids, anti-inflammatory medications, etc.) during the testing period. Before participation each subject gave written informed consent. The study was approved by the Departmental Human Ethics Committee following the principles outlined in the Declaration of Helsinki.

### Experimental Protocol

Prior to reporting to the laboratory, participants were asked to refrain from performing intense physical activity or consuming either caffeine or alcohol for a minimum of 24 hours prior to the trial and to maintain the same habitual routine for all trials. Each participant completed three trials as part of a randomized, cross-over design with a minimum of three days washout period between trials [7]. Participants reported to the laboratory at 0900 each trial day after an overnight (12 hr) fast. After quietly resting in an inclined-supine position for 15 min, an initial pre-ingestion capillary blood sample (95 μl) was obtained from an index finger and immediately analyzed for acid-base balance (ABL800 Basic analyzer, Radiometer, West Sussex, UK). Subsequently, the participants consumed 750 mL of water with either 9 g of fruit and vegetable concentrate (manufacturer recommendations from Energised Greens™ (EG), Nottingham, UK (Table 1)), 0.1 g·kg^-1 ^of NaHCO_3^- ^_(B) or a placebo (P) (plain flour) in opaque encapsulated pills within a 15 min period.

Once the 15 min ingestion period had completed, capillary samples were obtained and analyzed every 15 min thereafter for a period of 120 min. During this time, participants were also asked to rate any gastrointestinal (GI) discomfort they were experiencing using a visual analog scale (VAS). The VAS questionnaire has been used previously in the metabolic alkalosis literature [8], and is a commonly accepted tool for documenting subjective pain perception and discomfort [9].

### Statistical Analysis

All statistical analyses were completed using Statistica Software™ (Tulsa, OK) and GraphPad Prism 5.0™ (San Diego, CA). A two-way analysis of variance (ANOVA) with repeated measures (condition × time) were used to analyze differences in blood acid-base balance (pH, HCO_3^-^_, BE). GI discomfort (incidence & severity) data for each trial were analyzed using one-way ANOVA with repeated measures. Tukey's honestly significant difference (HSD) was performed in the event of a significant F ratio. Two-tailed statistical significance was accepted at p < 0.05. When significant differences are stated, the mean difference plus the 95% confidence interval (CI) of the mean difference are provided [10].

## Results

### Acid-Base Balance

**Figure 1:**
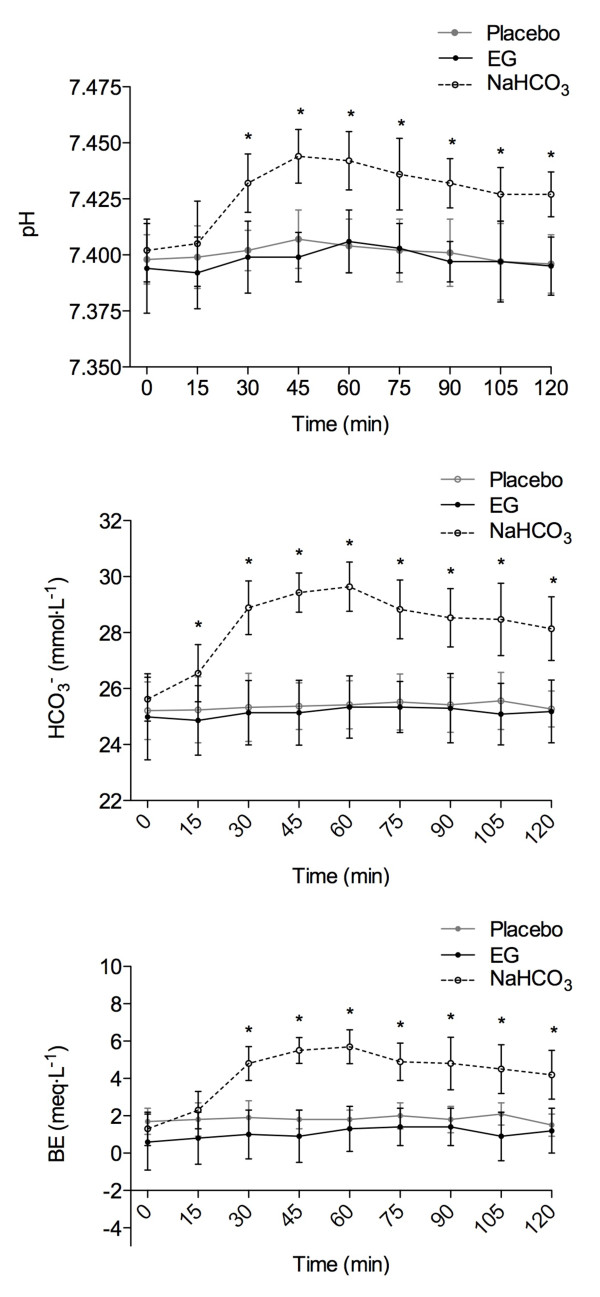
**Represented are the acid-base responses for Energised Greens™ (9 g) (EG), 0.1 g·kg^-1^BW sodium bicarbonate (NaHCO_3_) or flour placebo (Placebo) conditions over 120 min post ingestion**. For all three acid-base variables, only the NaHCO_3 _condition resulted in significant elevation (*) in blood alkalosis between 15 and 120 min (p < 0.01) when compared to both Placebo and EG.

There were significant interactions (p < 0.01) and main effects for condition (p < 0.001) and time (p < 0.001) for all acid-base variables (pH, HCO_3^-^_, & BE). Decomposition of the interactions indicated significant elevation in blood alkalosis for only the B condition when compared to both P and EG from 15 to 120 min during the ingestion period (Figure 1). Across this time frame, mean differences between pH for the B and EG trials were 0.013 (smallest) to 0.045 (largest) with 95%CI ranging between 0.01 to 0.07. This distribution was similar between the B and P trials (mean difference between 0.010 (smallest) to 0.040 (largest) with 95%CI ranging between 0.01 and 0.06). Following this profile, HCO_3^- ^_changes between B and EG trials ranged from the smallest mean difference of 1.6 mmol·L^-1 ^to the largest of 4.3 mmol·L^-1 ^(95%CI between 0.01 to 5.98 mmol·L^-1^), while B and P trials followed a similar pattern (smallest mean difference = 1.3 mmol·L^-1^; largest mean difference = 4.2 mmol·L^-1^; 95%CI between 0.4 to 5.9 mmol·L^-1^). Finally, base excess changes between the B and EG trials ranged from the smallest mean difference of 3.8 meq·L^-1 ^to the largest of 4.6 meq·L^-1 ^(95%CI between 0.13 to 6.24 meq·L^-1^), while B and P trials again were similar (smallest mean difference = 2.4 meq·L^-1^; largest mean difference = 3.9 meq·L^-1^; 95%CI between 0.7 to 5.5 meq·L^-1^).

### GI Discomfort

**Figure 2:**
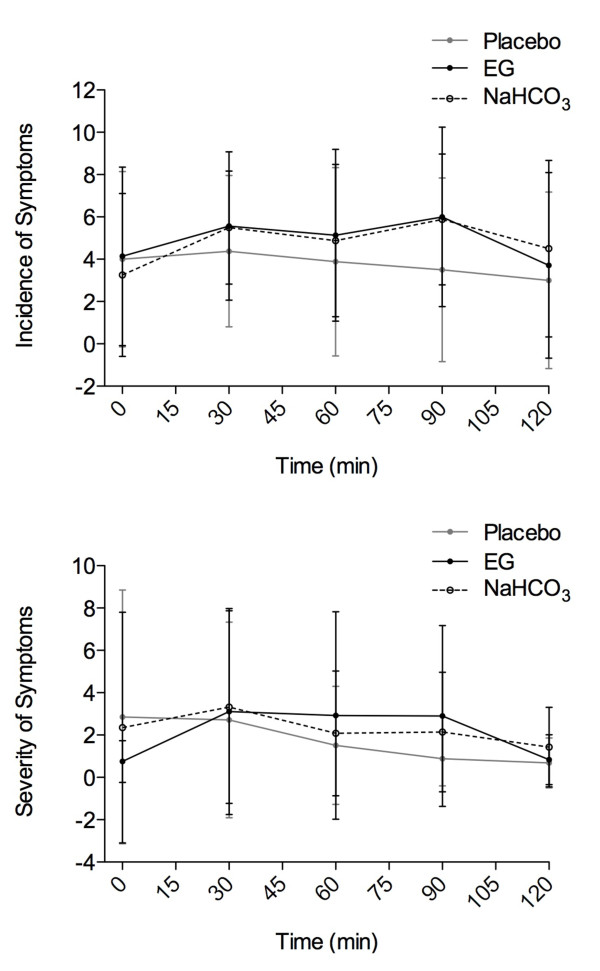
**Represented in the following figure are mean ± SD scores for both incidence and severity of symptoms over 120 minutes after ingestion of either Energised Greens™ (9 g) (EG), 0.1 g·kg^-1^BW sodium bicarbonate (NaHCO_3_) or flour placebo (Placebo)**.

A large degree of intra-subject variability was evident in both the incidence and severity of GI discomfort (Figure 2). There were no significant interactions (p > 0.98) or main effects for condition (p > 0.80) or time (p > 0.57) for either incidence or severity.

## Conclusions

The aim of the current investigation was to profile the differences in acid-base response following both acute fruit and vegetable extract (EG) consumption and a standard, low dose of sodium bicarbonate. Our findings suggest that acute EG supplementation only induces minimal blood alkalosis (Figure 1). Furthermore, these negligible acid-base alterations are not comparable to other acute alkalosis ingestion protocols (such as sodium bicarbonate ingestion), and therefore would presumably not provide additional buffering capacity during exercising conditions.

Our lack of an acute alkalotic shift in acid-base balance contrasts with other recently published work by König and colleagues [3]. These researchers presented significant increases in both blood and urine pH following acute multi-mineral supplementation in both males and females. The discrepancy between studies may illustrate the large variation between manufacturer recommendations on dosage administration levels and supplement contents (Table 1), as high concentrations of potassium contained within such supplements has shown to effect acid-base regulation to varying degrees [4]. Despite the high concentrations of metabolizing anions in fruits and vegetables in general and their purported role in absorption of H^+ ^[3], EG may not contain sufficient levels of pro-alkalizing nutrients to enhance blood-buffering capacity after a single ingestion [3,6].

As previously addressed, inducing acute increases in blood buffering capacity for performance enhancement via exogenous buffer ingestion often results in increased gastrointestinal (GI) distress [2,7]. An underlying aim of the current report was to not only use the NaHCO_3 _condition to compare acute blood buffering changes, but also to address the potential side-effect issue. Although our standard dose was on the low end of NaHCO_3 _doses [1,7], we felt that for a preliminary study this would be sufficient for comparison with the EG condition. Similar to other reports [2,8], we observed a large degree of variability between individuals for incidence and severity of symptoms between conditions (Figure 2). We acknowledge that this observation is based on a 0.1 g·kg^-1 ^and not a 0.3 g·kg^-1 ^NaHCO_3 _load, and that the GI distress reported in other studies in all likelihood resulted from the higher overall load of NaHCO_3_. However, we believe that future studies observing the chronic ingestion of EG do not need to consider GI distress in their methodologies.

In conclusion, acute ingestion of Energised Greens™ has only minor affects on blood acid-base regulation at rest and at 9 g would not induce sufficient changes in blood buffering capacity. Further research is warranted to investigate the potential chronic or dosage related loading effects of this product and other fruit and vegetable extracts upon blood acid-base regulation.

## Competing interests

The authors declare that they have no competing interests.

## Authors' contributions

MT was the principle investigator of the study. RP aided with data collection and analysis. MT, RP and JS conceived of the study, and participated in its design and coordination and helped to draft the manuscript. NM provided the supplements and proposed the idea of the study. All authors read and approved the final manuscript.
